# Plasmid-Mediated Ampicillin, Quinolone, and Heavy Metal Co-Resistance among ESBL-Producing Isolates from the Yamuna River, New Delhi, India

**DOI:** 10.3390/antibiotics9110826

**Published:** 2020-11-19

**Authors:** Mohammad Tahir Siddiqui, Aftab Hossain Mondal, Firdoos Ahmad Gogry, Fohad Mabood Husain, Ali Alsalme, Qazi Mohd. Rizwanul Haq

**Affiliations:** 1 Department of Biosciences, Jamia Millia Islamia, New Delhi 110025, India; tahir.sdq@gmail.com (M.T.S.); aftabmicro@gmail.com (A.H.M.); firdous071@gmail.com (F.A.G.); 2 Department of Food Science and Nutrition, College of Food and Agriculture Sciences, King Saud University, Riyadh 11451, Saudi Arabia; fahadamu@gmail.com; 3 Department of Chemistry, College of Science, King Saud University, Riyadh 11451, Saudi Arabia; aalsalme@ksu.edu.sa

**Keywords:** antibiotic resistance, plasmid-mediated quinolone resistance, AmpC β-lactamases, heavy metal resistance, aquatic environment

## Abstract

Antibiotic resistance is one of the major current global health crises. Because of increasing contamination with antimicrobials, pesticides, and heavy metals, the aquatic environment has become a hotspot for emergence, maintenance, and dissemination of antibiotic and heavy metal resistance genes among bacteria. The aim of the present study was to determine the co-resistance to quinolones, ampicillin, and heavy metals among the bacterial isolates harboring extended-spectrum β-lactamases (ESBLs) genes. Among 73 bacterial strains isolated from a highly polluted stretch of the Yamuna River in Delhi, those carrying *bla*CTX-M, *bla*TEM, or *bla*SHV genes were analyzed to detect the genetic determinants of resistance to quinolones, ampicillin, mercury, and arsenic. The plasmid-mediated quinolone resistance (PMQR) gene *qnrS* was found in 22 isolates; however, the *qnrA, B, C*, and *qnrD* genes could not be detected in any of the bacteria. Two variants of CMY, *bla*CMY-2 and *bla*CMY-42, were identified among eight and seven strains, respectively. Furthermore, *merB, merP, merT*, and *arsC* genes were detected in 40, 40, 44, and 24 bacterial strains, respectively. Co-transfer of different resistance genes was also investigated in a transconjugation experiment. Successful transconjugants had antibiotic and heavy metal resistance genes with similar tolerance toward antibiotics and heavy metals as did their donors. This study indicates that the aquatic environment is a major reservoir of bacteria harboring resistance genes to antibiotics and heavy metals and emphasizes the need to study the genetic basis of resistant microorganisms and their public health implications.

## 1. Introduction

Ever since their discovery, antibiotics have provided many benefits in fields ranging from healthcare to agriculture. However, the emergence of antibiotic resistance has significantly reduced their efficacy and usefulness in treating humans and animals. Emergence of antibiotic resistance among microorganisms is associated with many complex metabolic and genetic factors. Among them, extended spectrum β-lactamases (ESBLs), AmpC β-lactamases, and plasmid-mediated quinolone resistance (PMQR) genes are major factors in resistance to a variety of drugs [1,2,3]. ESBLs are one of the most important mechanisms providing resistance against large numbers of life-saving drugs. CTX-M, TEM, and SHV are the most common type of ESBLs identified in microorganisms [3,4]. Similarly, AmpC β-lactamases is a very important enzyme playing a crucial role in resistance development. Bacteria over expressing AmpC β-lactamases are generally resistant to large numbers of β-lactam antibiotics. CMY-1 was the first reported plasmid-mediated AmpC β-lactamase in 1989 [5]. Among a large number of variants, CMY-2 is the most common found among bacteria [6]. CMY-42 is another important variant of CMY-2 with increased activity against extended-spectrum antibiotics [7]. Plasmid-mediated transfer of AmpC β-lactamase genes is highly efficient in disseminating resistance to other microorganisms by horizontal gene transfer [8,9]. Bacterial strains carrying PMQR genes are among major factor in the decreasing efficacy of quinolones against human and animal infections [10]. PMQR genes are a set of six *qnr* genes (*qnrA, qnrB, qnrC, qnrD, qnrS*, and *qnrVC*) that encode gyrase-protection repetitive peptides [11,12], the aminoglycoside and quinolone-inactivating acetyl-transferase encoding *aac(6′)-Ib-cr* gene [11] and efflux pumps encoding genes *qaqBIII*, *oqxAB*, and *qepA* [13]. A number of PMQR genes, e.g., *qnrA, qnrS*, and *aac(6′)-Ib-cr* have been detected among clinically important bacteria from poultry and aquatic ecosystems [14,15,16].

In addition to antibiotics, other pollutants such as heavy metals, pesticides, and detergents also contribute significantly for the development of antibiotic resistance [17,18,19]. Countries like India are witnessing extensive industrial and urban development processes, which frequently cause the contaminations of surface water by heavy metals (e.g., mercury and arsenic) and other pollutants. The Delhi stretch of river Yamuna receives the pollutants from domestic, agriculture, and hospital discharges. Furthermore, a number of sewage and wastewater treatment plants also released the contamination to Yamuna. Studies on water and soil quality of Delhi stretch of river Yamuna showed a significantly high concentration of various antibiotics and heavy metals in river water including mercury and arsenic [20,21]. The maintenance of heavy metals like mercury and arsenic in aquatic environments could help bacteria in acquisition of metal resistance determinants located on the chromosome or plasmids [22]. Reports from the United States, the Netherlands, India, and Australia suggested the occurrence of heavy metal resistance among bacterial isolates [23,24,25,26]. Different resistance determinants are responsible for conferring resistance to heavy metals, e.g., mer and ars operon genes are responsible for resistance against mercury and arsenic, respectively [27]. It is also evident from reports that heavy metal resistance genes could be co-located on the same plasmids as those of antibiotic-resistance genes. Thus, the presence of heavy metals in the environment may exert a selective pressure towards bacteria carrying these mobile genetic elements [28,29]

Highly polluted aquatic ecosystems accommodate a diverse population of bacteria and further provide optimum conditions for development and propagation of resistance to antibiotics and heavy metals. Such aquatic environments could be termed hot spots of resistant microorganisms. ESBLs, AmpC, and PMQR are known to be responsible for resistance against a large number of crucial drugs for the treatment of life-threatening infections. Co-existence of these resistance mechanisms among bacteria from freshwater sources could be very concerning. However, there are only a few studies that report the co-occurrence of extended-spectrum β-lactamase (ESBLs), AmpC, PMQR, and heavy metal resistance determinants among water borne bacteria [30,31]. More studies in different settings and diverse geographical areas are needed for better information about antibiotics and heavy metal co-resistance. Thus, the present study was designed to identify the co-existence and co-transfer of PMQR, AmpC, mercury, and arsenic resistance determinants among bacterial strains carrying various ESBL genes from the Yamuna River, Delhi (India).

## 2. Materials and Methods

### 2.1. Features of the Included Bacterial Strains

**Figure 1:**
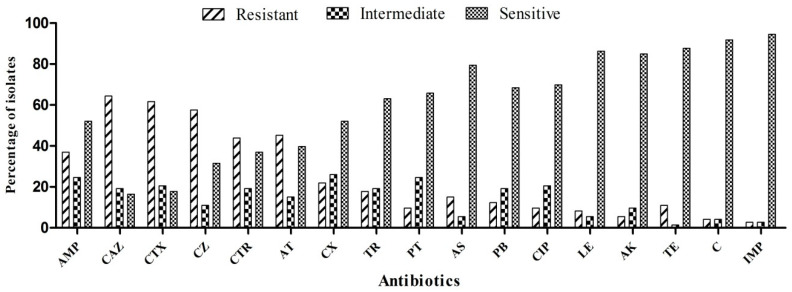
Antibiotic susceptibility pattern of extended-spectrum β-lactamases (ESBL) producing bacterial strains used in this study. Note 1: AMP, ampicillin; CAZ, ceftazidime; CTX, cefotaxime; CZ, cefazolin; CTR, ceftriaxone; AT, aztreonam; CX, cefoxitin; TR, trimethoprim; P/T-piperacillin/tazobactam, PB, polymyxin B; CIP, ciprofloxacin; A/S, ampicillin/sulbactam; LE, levofloxacin; AK, amikacin; TE, tetracycline; C, chloramphenicol; IPM-imipenem. Note 2: “Resistant”—Bacteria which could resist the effect of prescribed dosage concentration of antibiotic. “Intermediate”—Bacteria on its course to be resistant to prescribed concentration of antibiotic. “Sensitive”—Bacteria, which could not resist the prescribed dosage concentration of antibiotic.

The ESBL-producing bacterial stains (*n* = 73) harboring *bla*CTX-M and/or *bla*TEM, and/or *bla*SHV genes were selected for the present study from well-characterized bacteria previously reported by Siddiqui et al. [32]. All the ESBL-producing bacterial strains were isolated from the 10 different sites of highly polluted stretch of river Yamuna, Delhi, India (Supplementary Table S1). Bacterial strains were identified using amplification followed by sequencing and analysis of 16S rRNA gene. CTX-M, TEM, and SHV were presented among 73, 58, and 19 isolates respectively. Antibiotic resistance phenotype and genotype of ESBL-producing bacterial strains are shown in Supplementary Table S2 and Figure 1. All the cultures were preserved in 60% (*v/v*) glycerol at −80 °C. For subsequent experiments, bacterial strains were revived in Luria broth following overnight incubation and shaking at 37 °C and 120 rpm.

### 2.2. Antibiotic Susceptibility Testing and Multiple Antibiotic Resistance (MAR) Index

Antibiotic susceptibility testing of all the 73 test strains was repeated toward different classes of antibiotics using the Kirby Bauer disk diffusion technique as per Clinical and Laboratory Standards Institute guidelines (CLSI 2016). Seventeen different antibiotics used in the study included: amikacin (30 μg), ampicillin (10 μg), ampicillin/sulbactam (10/10 μg), piperacillin/tazobactam (100/10 μg), cefazolin (30 μg), cefoxitin (30 μg), ciprofloxacin (5 μg), levofloxacin (5 μg), imipenem (10 μg), polymyxin-B (300 units), aztreonam (30 μg), trimethoprim (5 μg), tetracycline (30 μg), chloramphenicol (30 μg), cefotaxime (30 μg), ceftazidime (30 μg), and ceftriaxone (30 μg) (all from HiMedia, India). After overnight incubation at 37 °C, zone of inhibition was measured, susceptibility pattern was determined, and bacterial strains were classified into resistant, intermediate, and sensitive with the help of CLSI guidelines. The multiple antibiotic resistance (MAR) index of each isolate was calculated as described by Krumperman [33]. The overall resistance of a single isolate towards 17 tested antibiotics is represented as multiple antibiotic resistance index (MAR Index). The MAR index is risk assessment tool that helps in differentiation of high- and low-risk region where antimicrobial drugs has been overused. An MAR index > 0.2 indicates the overuse of antimicrobial drugs and high selection pressure in the river, whereas MAR index < 0.2, indicates the low risk of source contamination in the river.

### 2.3. Plasmid DNA Isolation and Amplification

Plasmid DNA was extracted by the alkaline lysis method [34]. Extracted plasmid DNA was used as a template and PCR amplification was carried for all the genes using specific sets of primers (Table 1). PCR amplification of antibiotics and heavy metal resistance genes was carried out in a 50 μL reaction volume, comprising 5 μL of 10× buffer, 3 μL of 25 mM MgCl2, 1 μL of 10 mM dNTP mix, 1 μM of each forward and reverse primer, 2U of Taq-polymerase (Promega, Madison, WI, USA) and 2 μL (30 ng/μL) of template DNA. For each PCR, initial denaturation at 95 °C for 5 min was done, followed by 35 cycles of: denaturation (94 °C for 1 min), annealing (respective Tm of primer for 1 min), extension (72 °C for 1 min), and a final extension at 72 °C for 10 min. In order to check the positive amplification, PCR products were subjected to electrophoresis on 1% agarose gel (GENEI, Bengaluru, India). Gels were further visualized and photographed in a gel documentation system (BIO-RAD, Hercules, CA, USA). Amplicon sizes of different genes were checked using a 100 bp DNA molecular weight marker as reference (Fermentas, Waltham, MA, USA).

### 2.4. Detection and Characterization of blaCMY and PMQR Genes

All the bacterial strains were screened for the existence of plasmid-mediated *bla*CMY and PMQR (*qnrA, qnrB, qnrC, qnrD, qnrS*, and *aac(6′)-Ib*) genes with the help of specific sets of primers (Table 1). Purified PCR products were sequenced with corresponding gene primers (SciGenom Labs, Kochi, Kerala, India). Nucleotide sequences were analyzed by FinchTV software (Geospiza, Inc., Seattle, WA, USA), and the BioEdit program (http://www.mbio.ncsu.edu/bioedit/bioedit.html) was used to check homology of gene sequences with already available GenBank sequences. Variants of different genes were determined using the NCBI Basic Local Alignment Search Tool (BLAST) and homology analysis.

### 2.5. Detection of Heavy Metal Resistance Genes

All the test isolates were checked for the possible co-occurrence of heavy metal (mercury and arsenic) resistance genes. Occurrence of heavy metal resistance determinants were checked with plasmid DNA as a template and specific sets of primers (Table 1). Mercury resistance genes, e.g., *merB*, *merP*, and *merT*, were amplified by the protocol described by Azam et al. [40]. Similarly, the arsenic resistance gene *arsC* was amplified as per the method of Sunita et al. [25]. PCR products were run on a 1% agarose gel to determine positive amplification.

### 2.6. Susceptibility towards Heavy Metals

Heavy metal tolerance was recorded among bacterial strains harboring any mercury and/or arsenic resistance determinants. Minimum inhibitory concentration (MIC) of mercuric chloride (HgCl_2_) was determined against the strains carrying *mer* genes, whereas the MIC of sodium arsenate (Na_2_HAsO_4_) was recorded in bacteria positive for the *arsC* gene. Methods described by Sunita et al. and Figueiredo et al. were used to check the tolerance of bacteria against heavy metal concentrations [25,41]. Briefly, Serial dilutions (1 mg/L to 512 mg/L) of heavy metal salts were prepared in 96 well microtiter plate containing 200 μL of Luria broth. Medium was inoculated with 100 μL of 10,000 times diluted 0.1 OD_600_ culture of each isolate and incubated at 37 °C at 120 rpm for 14–18 h in an automated incubator shaker. The OD of samples was measured in a microplate reader (Thermo Scientific MultiskanGo, Waltham, MA, USA) at 600 nm wavelength, and MIC was determined. *E. coli* ATCC 25922 strains was used as negative control. Media without heavy metal salt was also served as control.

### 2.7. Conjugation Experiment

A conjugation experiment was carried out to determine the transferability of antibiotic and metal resistance genes (*bla*CTX-M, *bla*TEM, *bla*SHV, *bla*CMY, PMQR, *merB*, *merP*, *merT*, and *arsC*). Seven different isolates, namely, *E. coli* MKT3, *Acinetobacter junii* IBT29, *Enterobacter cloacae* BVT29, *E. coli* MBT29, *K. pneumoniae* MBT50, *E. coli* CCT42, and *Acinetobacter* sp. BOT40, harboring antibiotic and heavy metal resistance determinants were chosen as donors. Plasmid-free *Escherichia coli* J53 strain resistant to sodium azide and sensitive to antibiotics and heavy metal used in this study was selected as recipient. Transconjugation experiment was performed by the help of protocol described by Zurfluh et al. [31]. In brief, secondary cultures of donor and recipient bacterial isolates were mixed in a 1:1 ratio in Luria–Bertani broth (HiMedia, Mumbai, India) and incubated at 37 °C for 24 h without any shaking. Serial dilutions of transconjugant bacteria were streaked on LA plates containing cefotaxime (2 μg/mL) and sodium azide (100 μg/mL). After overnight incubation, transconjugant colonies were selected and revived for the plasmid isolation. PCR-based amplification using transconjugant plasmids as templates was carried out to detect the successful transfer of antibiotic and heavy metal resistance determinants.

MIC of ampicillin, ciprofloxacin, cefotaxime, mercuric chloride (HgCl_2_), and sodium arsenate (Na_2_HAsO_4_) against donors and transconjugants were further determined.

### 2.8. Accession Numbers

The DNA sequences of the *qnrS* gene were deposited in the GenBank database under accession numbers MH426935-MH426956 (No. 22). Complete gene sequences of *bla*CMY-2 and *bla*CMY-42 were deposited under the accession numbers MH426957-MH426964 (No. 8) and MH426965-MH426971 (No. 7).

## 3. Results

The present study was aimed to study the co-occurrence of PMQR, *bla*CMY, and heavy metal resistance genes among bacterial strains harboring ESBL genes collected from the highly polluted Yamuna River. Detailed results of this work are discussed below.

### 3.1. Susceptibility Profile of Test Strains

All the 73 bacterial strains were exposed to 17 different antibiotics to analyze their susceptibility patterns by the help of CLSI-2016 guidelines and classified into resistant, intermediate, and susceptible strains. Terms resistant, intermediate, and sensitive have been recommended by the CLSI (clinical laboratory standard institute) for the classification of bacteria on the basis of antibiotic susceptibility. “Resistant” are the organisms, which could resist the effect of prescribed dosage concentration of antibiotics, “intermediate” are those bacteria, which are on the course to be resistant to prescribed drug concentration, and “sensitive” are those, which could not resist the prescribed concentration of antibiotics. Analysis of antibiotic profiling found that the majority of test strains were resistant to ceftazidime (62/73), followed by cefotaxime (59/73), cefazolin (50/73), ceftriaxone (46/73), ampicillin (45/73), aztreonam (44/73), cefoxitin (35/73), trimethoprim (28/73), piperacillin/tazobactam (25/73), polymyxin B (23/73), ciprofloxacin (22/73), ampicillin/sulbactam (15/73), levofloxacin (11/73), amikacin (11/73), tetracycline (10/73), chloramphenicol (6/73), and imipenem (4/73) (Supplementary Table S2). Antimicrobial susceptibility patterns of all the tested strains are shown in Figure 1. The multiple antibiotic resistance (MAR) index of different bacteria was found to be in the range of 0.941–0.058. The MAR index of all tested bacterial strains is shown in Table 2.

### 3.2. Detection and Characterization of blaCMY and PMQR Genes

Alkaline lysis method was used to extract the plasmid DNA from all the tested bacterial strains. In order to confirm the presence of plasmid, extracted genetic material from all the strains were subjected to 16S rRNA gene amplification, since this gene is known to be located on chromosomal DNA, it does not amplify by the plasmid as a template. Non-amplification of 16S rRNA gene confirmed the presence of plasmid DNA among 73 tested bacterial isolates.

Of 73 bacterial strains, 15 (20.5%) harbored the *bla*CMY gene. Nucleotide sequence analysis revealed the presence of two variants, *bla*CMY-2 and *bla*CMY-42. *bla*CMY-2 was found and characterized among eight strains, whereas *bla*CMY-42 was identified in seven bacteria (Table 2). Furthermore, PMQR genes (*qnrA, qnrB, qnrC, qnrD, qnrS*, and *aac(6′)-Ib*) were amplified and only *qnrS* was detected among 22 bacterial strains. Nucleotide sequences of all the 22 amplified products were analyzed for their homology with previously submitted sequences and submitted to NCBI GenBank database. The *qnrA, qnrB, qnrC, qnrD*, and *aac(6′)-Ib* genes could not be detected among any of the bacterial strains. The distribution pattern of antibiotic resistance genes is presented in Table 2.

### 3.3. Detection of Heavy Metal Resistance Genes

**Figure 2:**
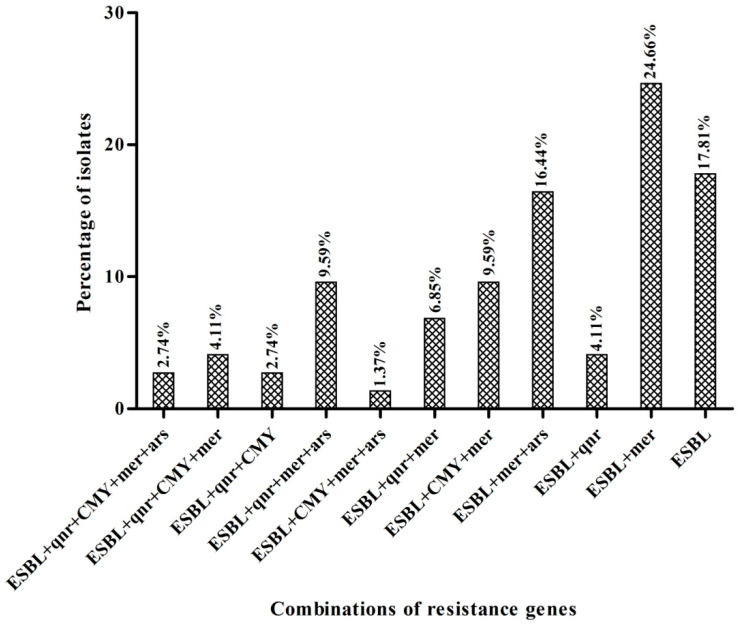
Percentage of bacterial strains harboring different resistance genes.

All the 73 bacterial strains were checked for the presence of heavy metal resistance genes on their plasmid DNA. Mercury resistance genes *merB, merP*, and *merT* were detected among 40, 40, and 43 strains, respectively (Table 2). Furthermore, the arsenic resistance gene *arsC* was successfully amplified among 24 strains (Table 2). Distribution patterns of heavy metal resistance genes among test strains are shown in Table 2. Combinations of different antibiotics and heavy metals resistance genes are presented in Figure 2.

### 3.4. Susceptibility to Heavy Metals

All the bacterial strains carrying any of the respective metal resistance genes were studied to determine their tolerance toward HgCl_2_ or Na_2_HAsO_4_.Isolates harboring any of the *mer* genes showed tolerance to HgCl_2_ in the range of <1 to 8 mg/L. The MIC of Na_2_HAsO_4_ was in the range of 16 to >512 mg/L against the strains carrying the *arsC* gene (Table 2).

### 3.5. Conjugation Experiments

In vitro conjugation experiments were carried out with seven strains, *E. coli* MKT3, *Acinetobacter junii* IBT29, *Enterobacter cloacae* BVT29, *E. coli* MBT29, *K. pneumoniae* MBT50, *E. coli* CCT42, and *Acinetobacter* sp. BOT40, all harboring maximum numbers of antibiotic and heavy metal resistance genes. Transconjugants were successfully obtained on cefotaxime-supplemented Luria agar plates. Conjugation efficiencies were recorded for *E. coli* MKT3, *Acinetobacter junii* IBT29, *Enterobacter cloacae* BVT29, *E. coli* MBT29, *K. pneumoniae* MBT50, *E. coli* CCT42, and *Acinetobacter* sp. BOT40 transconjugant as 3.9 × 10^−3^, 2.1 × 10^−3^, 2.6 × 10^−3^, 5 × 10^−3^, 2.1× 10^−3^, 6.7× 10^−3^, and 1.6 × 10^−2^, respectively. Plasmid analysis of recipient *E. coli* J53AZ^R^ revealed that donor isolates were able to co-transfer the antibiotic and heavy metal resistance determinates such as CTX-M, TEM, *qnrS*, CMY, and *mer* via conjugation (Table 3). The *arsC* gene could not be detected in any of the transconjugants. The MIC of ampicillin, cefotaxime, ciprofloxacin, HgCl_2,_ and Na_2_HAsO_4_ was determined against transconjugants and was similar to that of their donor bacterial strains (Table 3).

## 4. Discussion

In the present study, we investigated the presence of PMQR, *bla*CMY, and heavy metal resistance genes among bacterial strains harboring ESBL genes collected from the highly polluted Yamuna River [32]. The most important finding of this work was co-occurrence of antibiotic and heavy metal resistance determinants among bacterial strains and their co-transfer to the host strain.

Our work highlights the importance of the resistance gene pool and its interaction with diverse groups of bacteria in the urban aquatic environment. In the present work, we studied the co-existence of PMQR, *AmpC*, and heavy metal resistance determinants among bacterial strains of aquatic origin harboring ESBL genes. The horizontal transfer of different resistance genes was also determined with conjugation experiments. One of the very important aspects of this study was the co-occurrence of different antibiotic and heavy metal resistance determinants among tested bacterial strains of aquatic origin.

Domestic, hospital, municipal, industrial and agricultural wastes continuously increase the pollution level of urban aquatic bodies. This high degree of pollution creates selection pressure for the emergence, dissemination, and maintenance of antibiotic and heavy metal resistance among aquatic microorganisms. Studies on water and soil quality of the Delhi stretch of the Yamuna River showed a significantly high concentration of various antibiotics and heavy metals in river water [20,21]. Furthermore, the incidence of antibiotic and heavy metal resistance determinants among microorganisms from polluted aquatic environments is significant [9,32,42,43,44].

In the present study, our test strains had a very high level of resistance to β-lactam antibiotics. Antibiotic profiling of 73 tested bacterial strains showed elevated resistance toward ceftazidime (84.9%), followed by cefotaxime (80.8%), cefazolin (68.4%), ceftriaxone (63%), ampicillin (61.6%), aztreonam (60.2%), and cefoxitin (47.9%). However, bacterial strains were found to be very sensitive to imipenem (5.4% resistance). A similar resistance pattern for β-lactam antibiotics was recorded by Lu et al. from an urban river habitat in China [45]. Moreover, our results are also in line with other studies reporting a high level of resistance against β-lactam and non-β-lactam antibiotics [46,47,48,49,50,51,52]. This study found 34.2% and 20.5% resistance toward piperacillin + tazobactam and ampicillin + sulbactam combinations, respectively. A study by Lu et al. also found similar resistance patterns against combinations of antibiotic and β-lactamase inhibitors among ESBL producers [45]. A varied resistance pattern was found against non-β-lactam antibiotics, e.g., trimethoprim (38.5%), polymyxin B (31.5%), ciprofloxacin (30.1%), levofloxacin (15%), amikacin (15%), tetracycline (13.6%), and chloramphenicol (8.2%). Our results correspond with those in a study on hospital wastewater in Vietnam, which showed similar resistance patterns to antimicrobials [53]. The MAR index of each bacterial strain used in the study was determined and was in the range of 0.941–0.058. We found the MAR index greater than 0.2 among 84.9% of strains. MAR index values greater than 0.2 suggests a high-risk source of contamination [33,47,54].

Plasmid-mediated CIT type *AmpC* β-lactamase was successfully amplified among 20.5% of bacterial strains. Bajaj et al., Martinez-Medina et al., and Loncaric et al. also reported the similar occurrence of *AmpC*-type β-lactamase among bacterial isolates from river water and patient samples, respectively [9,52,55]. Furthermore, sequence analysis revealed the occurrence of *bla*CMY-2 and *bla*CMY-42 among 8 and 7 strains, respectively. Among CIT type *AmpC* β-lactamases, *bla*CMY-2 is one of the most common variants reported in various studies [56,57]. *bla*CMY-42 is one of the variants of *bla*CMY-2 evolved by substitution of *val231Ser* [7,9]. Presence of the *bla*CMY-42 gene among aquatic isolates is a serious issue because it confers a high level of resistance against third-generation cephalosporins and monobactams.

Quinolone and β-lactam antibiotics are very common drugs for the treatment of various infections. Thus, the co-occurrence of resistance toward both of these drugs could pose a serious public health concern [14,15]. PMQR genes are major contributors in conferring resistance toward fluoroquinolone drugs [10,11]. Six different PMQR genes (*qnrA, qnrB, qnrC, qnrD, qnrS*, and *aac(6′)-Ib*) were checked for their occurrence among bacterial strains with ESBL genes. Successful amplification of the *qnrS* gene was observed among 30.1% of tested strains. None of the isolates was positive for *qnrA, qnrB, qnrC, qnrD*, and *aac(6′)-Ib*. Sequence homology analysis of partial genes revealed 100% similarity with the already available *qnrS* gene in the NCBI GenBank database. Our results are in accordance with the study of Bajaj et al., where 50% of CTX-M-producing isolates were positive for the *qnrS* gene [14]. Co-occurrence of PMQR and other antibiotic resistance genes has been reported in similar studies from China, Iran, and Austria [55,58,59]. The presence of PMQR genes among bacteria is a cause for concern, because these resistance genes are thought to create an optimum environment for selection of chromosomal resistance determinants [60,61]. This could pose a further serious health risk to human and animal populations. It is imperative to increase the scope of surveillance studies of drug-resistant bacteria to the genotypic level so that identification of the cause of resistance can be determined accurately.

Occurrence of mercury resistance genes (*merB, merP* and *merT*) and the arsenic resistance gene (*arsC*) was determined among ESBL-positive strains. In the present study, we observed the presence of *merB*, *merP*, and *merT* genes among 54.7%, 54.7%, and 58.95% of tested bacteria, respectively. The genes *merB, merP*, and *merT* are known to play a crucial role in the emergence of mercury resistance, but the combined action of all the genes of the *mer* operon exerts broad-spectrum resistance among microorganisms [62]. Studies among environmental bacteria from the United States and Brazil also reported the presence of *mer* operon genes in significantly high percentages [63,64]. Co-occurrence of antibiotic and mercury resistance gene was also reported by many studies carried out in India and Australia [40,65]. All the strains positive for at least one *mer* gene were tested for their tolerance toward mercuric chloride. The MIC of mercuric chloride was found in the range of <1 to 8 mg/L. Azam et al. and Rebello et al. also reported a similar tolerance pattern among bacterial isolates of river origin [40,64]. Various genes are known to be responsible for conferring arsenic resistance among bacteria. *arsC* is one of the key genes determining arsenic resistance by reducing arsenate to arsenite [66]. In this study, we observed the occurrence of the *arsC* gene on the plasmid DNA of 32.8% of bacteria. Di Cesare et al. reported the occurrence of *arsC* genes among antibiotic-resistant bacteria obtained from wastewater in Italy [43]. Other studies among bacterial isolates of environmental origin also showed the presence of the *arsC* gene in abundance [25,43,67]. In determining the MIC of sodium arsenate against the isolates harboring the *arsC* gene, we found that bacterial isolates were able to tolerate 16 to > 512 mg/L concentration of sodium arsenate. Studies on arsenic-tolerant bacteria carried out in India and Indonesia also showed the similar MICs of sodium arsenate [68,69]. Our results are in agreement with the previous studies on characterization of the *arsC* gene among bacteria in India and Italy [25,67,70].

We performed a conjugation experiment to check the possible horizontal transfer of antibiotic and heavy metal resistance genes. An in vitro conjugation experiment in this study revealed that bacterial strains *E. coli* MKT3, *Acinetobacter junii* IBT29, *Enterobacter cloacae* BVT29, *E. coli* MBT29, *K. pneumoniae* MBT50, *E. coli* CCT42, and *Acinetobacter* sp. BOT40 successfully transferred antibiotic and heavy metal-resistant genes to recipient *E. coli* J53AZ^R^. We did not find the presence of *arsC* gene in any of the transconjugants, which showed the possibility of presence of this gene on other non-conjugative plasmid. This result of conjugation experiment suggests the possibility of resistance gene transfer between different bacteria in environmental settings. The transfer of resistance determinants in the aquatic environment could significantly enhance the opportunity for pathogens to acquire resistance [71]. Our data corroborate those in the study conducted by Liu et al., in which successful transfer of various drug resistance genes was carried out through transconjugation [72]. A study conducted among bacterial isolates from wastewater in Italy has also suggested a co-selection of resistance genes of antibiotics and heavy metals [43]. Because antibiotic and heavy metal resistance genes are known to be located on a similar gene cluster, and also on the same conjugative plasmids, it is imperative to further explore the possible mechanisms of heavy metal-driven emergence and dissemination of drug resistance.

## 5. Conclusions

Waterborne bacterial strains carrying ESBL genes were found to harbor resistance determinants for *AmpC* β-lactamase, PMQR, mercury resistance, and arsenic resistance. The presence of resistance genes for such a large number of antimicrobial agents is a serious cause for concern. Detection of *bla*CMY-2 and *bla*CMY-42 is also very significant because these determinants confer increased resistance against cephalosporins and monobactams. Furthermore, coexistence and plasmid-mediated co-transfer of antibiotic and heavy metal resistance genes is a very serious issue. It warrants the deeper and large-scale study on molecular mechanisms of these resistance genes in different settings. Moreover, it is also very important to unravel the role of environmental factors influencing the emergence and dissemination resistance among microorganisms. Study also emphasizes the stern need of regulation on the use of various antimicrobial and heavy metal substances, which helps in the creation of selection pressure responsible for the rise of resistance.

## Figures and Tables

**Table 1:** Primers used for amplification of different genes.

Gene	Primer Sequence	Product Size	References
***merB***	5′-ATGAAGCTCGCCCCATATATTTTA-3′ 5′-TCACGGTGTCCTAGATGACATGGT-3′	640 bp	This study
***merP***	5′-ATGAAGAAACTGTTTGCCTCCCTT-3′ 5′-TCACTGCTTGACGCTGGACG GA-3′	272 bp	This study
***merT***	5′-TTAATAGAAAAATGGAACGACATA-3′ 5′-ATGTCTGAACCACAAAACGGG CG3′	355 bp	This study
***arsC***	5′-GTAATACGCTGGAGATGATCCG-3 5′-TTTTCCTGCTTCATCAACGAC-3′	409 bp	[25]
***qnrA***	5′-TGTATTTCGCTGTTGCTGGGGAG-3′ 5′-AAGTGACCAGAATAAGCGGC-3′	580 bp	[15]
***qnrB***	5′-ATG GTG ACA AAG AGA GTG CA-3′ 5′-GTT CTG TTG CGG CTG GGT AA -3′	476 bp	[35]
***qnrC***	5′-GGGTTGTACATTTATTGAATC-3′ 5′-TCCACTTTACGAGGTTCT-3′	447 bp	[36]
***qnrD***	5′-CGAGATCAATTTACGGGGAATA-3′ 5′-AACAAGCTGAAGCGCCTG-3′	582 bp	[37]
***qnrS***	5′-GCCTGTGTTTCGCTGCTGTT-3′ 5′-AGTAAGTCACCAGAACGAGC-3′	428 bp	[15]
***aac(6′)-Ib***	5′-TTGCGATGCTCTATGAGTGGCTA-3′ 5′-CTCGAATGCCTGGCGTGTTT-3′	482 bp	[38]
***bla*CMY**	5′-ATGAGTATTCAACATTTCCG-3′ 5′-CCAATGCTTAATCAGTGAGG-3′	1226 bp	[39]

**Table 2:** Antibiotic and heavy metal resistance genes among tested bacterial isolates and their corresponding minimum inhibitory concentrations (MICs).

Strains	ESBLs Genes (from Previous Study)	MAR Index	PMQR Gene	*bla*CMY Gene	Heavy Metal Resistance Genes				MIC	
					*merB*	*merP*	*merT*	*arsC*	HgCl_2_ (mg/L)	Na_2_HAsO_4_ (mg/L)
*Acinetobacter calcoaceticus* SRT58	CTX-M-15	0.588	*qnrS*		-	+	-	-	<1	-
*Acinetobacter calcoaceticus* CCT66	CTX-M-15	0.647			-	-	-	-	-	-
*Acinetobacter johnsonii* BOT41	CTX-M-152	0.411			-	-	-	-	-	-
*Acinetobacter schindleri* BOT46	CTX-M-15	0.235			+	+	+	-	<1	-
*Acinetobacter schindleri* CCT65	CTX-M-15, CTX-M-152, TEM-116	0.294		CMY-2	-	+	+	-	2	-
*Acinetobacter* sp. BOT44	CTX-M-152, TEM-116	0.352			-	-	-	-	-	-
*Acinetobacter* sp. BOT40	CTX-M-15, TEM-116	0.529		CMY-2	+	-	+	+	2	>512
*Acinetobacter* sp. CCT53	CTX-M-15, CTX-M-152, TEM-116	0.176			+	+	+	-	8	-
*Acinetobacter* sp. CCT54	CTX-M-15, TEM-116	0.058			+	+	+	+	4	>512
*Acinetobacter* sp. CCT59	CTX-M-15, CTX-M-152 ‘TEM-181	0.235			-	-	-	-	-	-
*Acinetobacter* sp. MKT43	CTX-M-15, TEM-116	0.588	*qnrS*		+	+	+	-	8	-
*Acinetobacter* sp. MKT45	CTX-M-15, CTX-M-152, SHV-12	0.529	*qnrS*		-	+	-	-	<1	-
*Acinetobacter* sp. MKT46	CTX-M-15	0.235			-	+	+	-	1	-
*Acinetobacter* sp. MKT48	CTX-M-15, CTX-M-152, SHV-42	0.411		CMY-2	+	+	+	-	8	-
*Acinetobacter* sp. SRT63	CTX-M-152, TEM-116	0.117			+	-	+	-	<1	-
*Acinetobacter* sp. SRT64	CTX-M-3	0.529		CMY-42	-	+	-	-	<1	-
*Acinetobacter* sp. SRT65	CTX-M-15	0.529	*qnrS*		+	-	+	-	1	-
*Acinetobacter* sp. RBT26	CTX-M-15, CTX-M-152, TEM-116	0.470		CMY-2	+	+	+	-	<1	-
*Acinetobacter junii* IBT27	CTX-M-15	0.294			+	+	+	+	4	256
*Acinetobacter junii* IBT29	CTX-M-15, TEM-116	0.705	*qnrS*	CMY-42	-	+	+	-	4	-
*Acinetobacter junii* IBT30	CTX-M-15, CTX-M-152, TEM-116	0.294			-	+	-	-	<1	-
*Acinetobacter junii* IBT31	CTX-M-15, CTX-M-152	0.235			-	+	-	-	<1	-
*Acinetobacter junii* IBT33	CTX-M-15, CTX-M-152, TEM-141, SHV-12	0.294	*qnrS*		+	+	+	+	1	64
*Acinetobacter junii* IBT36	CTX-M-15, TEM-116	0.117			+	+	+	-	2	-
*Acinetobacter junii* IBT39	CTX-M-15	0.529		CMY-2	+	+	+	-	2	-
*Acinetobacter junii* IBT40	CTX-M-15, CTX-M-152, TEM-116	0.117			-	-	-	-	-	-
*Acinetobacter junii* RBT31	CTX-M-15, CTX-M-152, SHV-12	0.352			+	+	+	-	4	-
*Bacillus altitudinis* BOT30	CTX-M-15, TEM-116	0.176			+	-	+	-	<1	-
*Bacillus firmus* BOT39	CTX-M-15, CTX-M-152	0.235			-	-	+	-	<1	-
*Bacillus firmus* MBT57	CTX-M-15, TEM-116	0.352			-	+	-	-	1	-
*Bacillus firmus* WBT16	CTX-M15, TEM-116	0.235			+	-	+	-	<1	-
*Bacillus safensis* MKT35	CTX-M-15, TEM-116	0.235			+	+	+	-	8	-
*Bacillus safensis* BVT32	CTX-M-152, TEM-116	0.295			+	+	+	+	4	>512
*Brachymonas chironomi* MKT44	CTX-M-15, CTX-M-152, TEM-116, SHV-12	0.529	*qnrS*	CMY-42	+	+	+	-	8	-
*E. coli* MKT3	CTX-M-15, TEM-116, SHV-12	0.411	*qnrS*	CMY-42	-	+	-	+	8	>512
*E. coli* MKT25	CTX-M-15, TEM-116	0.588	*qnrS*		-	+	-	+	4	>512
*E. coli* BVT8	CTX-M-15, TEM-116	0.529	*qnrS*		-	-	-	-	-	-
*E. coli* BVT20	CTX-M-15, SHV-12	0.880	*qnrS*		+	-	-	+	<1	128
*E. coli* RBT1	CTX-M-15	0.705			+	+	+	-	2	-
*E. coli* IBT13	CTX-M-15, TEM-116, SHV-12	0.941	*qnrS*		+	-	+	+	2	64
*E. coli* SRT41	CTX-M-15, TEM-116	0.470		CMY-2	+	-	+	-	1	-
*E. coli* MBT16	CTX-M-15	0.529	*qnrS*	CMY-2	-	-	-	-	-	-
*E. coli* MBT29	CTX-M-15	0.880	*qnrS*	CMY-42	+	+	-	+	2	32
*E. coli* MBT42	CTX-M-15, TEM-116, SHV-12	0.176			+	-	-	+	<1	64
*E. coli* CCT7	CTX-M-15, TEM-116	0.880	*qnrS*		-	-	-	-	-	-
*E. coli* CCT42	CTX-M-15, TEM-116	0.823	*qnrS*		+	-	+	+	4	512
*E. coli* CCT43	CTX-M-15, TEM-116	0.176			-	-	-	-	-	-
*E. coli* CCT50	CTX-M-15	0.529			-	+	+	+	8	>512
*E. coli* CCT64	CTX-M-15, CTX-M-152, TEM-116	0.176			+	+	+	+	4	128
*E. coli* BOT1	CTX-M-15	0.235			-	-	-	-	-	-
*Enterobacter cloacae* BVT22	CTX-M-15, TEM-116, SHV-12	0.352			-	-	-	-	-	-
*Enterobacter cloacae* BVT29	CTX-M-15, CTX-M-152, TEM-116, SHV-12	0.705	*qnrS*		+	+	+	+	8	>512
*Enterobacter cloacae* BVT34	CT-M-15, TEM-116	0.235			-	-	-	-	-	-
*Enterobacter cloacae* GCT36	CTX-M-15	0.647		CMY-42	-	-	-	+	-	256
*Enterobacter cloacae* MBT49	CTX-M-15, CTX-M-152, TEM-116	0.294			-	-	-	-	-	-
*Enterobacter cloacae* CCT13	CTX-M-15	0.764	*qnrS*		-	-	-	-	-	-
*Enterobacter* sp. BVT30	CTX-M-5	0.235			+	+	+	+	8	512
*Enterobacter* sp. BOT45	CTX-M-15, CTX-M-152	0.176			+	+	+	+	1	>512
*K. pneumoniae* RBT40	CTX-M-15, CTX-M-152	0.294			+	+	+	+	8	>512
*K. pneumoniae* MBT43	CTX-M-15, TEM-116	0.235			+	-	+	-	4	-
*K. pneumoniae* MBT50	CTX-M-15	0.705	*qnrS*	CMY-42	+	-	+	-	2	-
*K. pneumoniae* MBT51	CTX-M-15, TEM-116	0.235			+	+	+	+	4	>512
*K. pneumoniae* MBT52	CTX-M-15, SHV-12	0.294			+	+	+	-	8	-
*K. pneumoniae* MBT59	CTX-M-15, CTX-M-152, TEM-116	0.117	*qnrS*		+	+	+	-	2	-
*K. quasipneumoniae* MKT39	CTX-M-3, TEM-116	0.470			+	-	+	-	<1	-
*Kluyvera georgiana* CCT51	CTX-M-15	0.235			+	+	+	+	8	>512
*Kluyvera georgiana* CCT69	CT-M-152, TEM-116	0.588			-	-	-	-	-	-
*Shigella flexneri* RBT20	CTX-M-15, TEM-116	0.705	*qnrS*		-	+	+	+	1	16
*Shigella flexneri* IBT7	CTX-M-15, TEM-116	0.705	*qnrS*	CMY-2	-	-	-	-	-	-
*Shigella sonnei* MKT34	CTX-M-15, TEM-116	0.176			-	-	-	+	-	64
*Shigella flexneri* CCT27	CX-M-15, TEM-116, SHV-12	0.235			-	-	-	-	-	-
*Shigella flexneri* CCT52	CTX-M-15, CTX-M-152	0.235			+	+	+	-	8	-
*Serratia marcescens* RBT37	CTX-M-15, TEM-116	0.294			+	+	+	+	8	>512

**Table 3:** Distribution of antibiotics and heavy metal resistance genes among transconjugants and their Minimum inhibitory concentrations (MIC)s.

Bacterial Strains	Resistance Genes									MIC				
	CTX-M	TEM	SHV	*qnrS*	CMY	*merB*	*merP*	*merT*	*arsC*	Ampicillin (µg/mL)	Ciprofloxacin (µg/mL)	Cefotaxime (µg/mL)	HgCl_2_ (mg/L)	Na_2_HAsO_4_ (mg/L)
*E. coli* MKT3	+	+	+	+	+	−	+	−	+	>1024	64	>1024	8	>512
Transconjugant (J53AZ^R^)	+	+	−	+	−	−	+	−	−	512	18	1024	2	32
*Acinetobacter junii* IBT29	+	+	−	+	+	−	+	+	−	128	32	256	4	64
Transconjugant (J53AZ^R^)	+	+	−	+	+	−	+	−	−	32	16	256	2	32
*Enterobacter cloacae* BVT29	+	+	+	+	−	+	+	+	+	256	32	256	8	>512
Transconjugant (J53AZ^R^)	+	+	+	+	−	+	+	+	−	256	8	32	8	32
*E. coli* MBT29	+	−	−	+	+	+	+	−	+	>1024	>1024	>1024	2	32
Transconjugant (J53AZ^R^)	+	−	−	+	+	−	−	−	−	256	256	512	1	32
*K. pneumoniae* MBT50	+	−	−	+	+	+	−	+	−	128	16	512	2	32
Transconjugant (J53AZ^R^)	+	−	−	+	+	−	−	+	−	128	8	512	1	32
*E. coli* CCT42	+	+	−	+	−	+	−	+	+	>1024	256	>1024	4	512
Transconjugant (J53AZ^R^)	+	+	−	−	−	+	−	−	−	512	64	256	2	32
*Acinetobacter* sp. BOT40	+	+	−	−	+	+	−	+	+	32	8	128	2	>512
Transconjugant (J53AZ^R^)	+	+	−	−	+	−	−	+	−	32	4	128	2	32

Note: *Escherichia coli* J53 strain used as recipient for transconjugation was sensitive to all the tested antibiotics and heavy metal in this study.

## Supplementary Materials

The following are available online at https://www.mdpi.com/2079-6382/9/11/826/s1, Table S1: Water samples collection sites of river Yamuna from where bacterial strains were isolated, Table S2: Bacterial strains used in this study with corresponding ESBLs genes and their antibiotics profiling.
