# Supplementary material for: Plasmid-Mediated Ampicillin, Quinolone, and Heavy Metal Co-Resistance among ESBL-Producing Isolates from the Yamuna River, New Delhi, India
Source: Antibiotics (Basel). 2020 Nov 19;9(11):826. doi: 10.3390/antibiotics9110826 (PMC7699290; doi:10.3390/antibiotics9110826)
Supplement: Supplementary file 1 [file antibiotics-09-00826-s001.pdf]

antibiotics

Plasmid-Mediated Ampicillin, Quinolone, and Heavy Metal Co-resistance among ESBL-Producing Isolates from the Yamuna River, New Delhi, India

Mohammad Tahir Siddiqui[1], Aftab Hossain Mondal[1], Firdoos Ahmad Gogry[1], Fohad Mabood Husain[2], Ali Alsalme[3] and Qazi Mohd. Rizwanul Haq[1,*]

[1] Department of Biosciences, Jamia Millia Islamia, New Delhi 110025, India; tahir.sdq@gmail.com (M.T.S.); aftabmicro@gmail.com (A.H.M.); firdous071@gmail.com (F.A.G.)

[2] Department of Food Science and Nutrition, College of Food and Agriculture Sciences, King Saud University, Riyadh 11451, Saudi Arabia; fahadamu@gmail.com

[3] Department of Chemistry, College of Science, King Saud University, Riyadh 11451, Saudi Arabia; aalsalme@ksu.edu.sa

* Correspondence: qhaque@jmi.ac.in, haqqmr@gmail.com; Tel.: +91-11-2698-1717 (ext. 74302); Fax: +91-11-2698-0229

Supplementary table 1- Water samples collection sites of river Yamuna from where bacterial strains were isolated (Siddiqui et al 2019).

S. No	Site Code	Sampling site	Latitude	Longitude
1.	WBT	Wazirabad Barrage	28°42’40.0212”N	77°13’51.2436”E
2.	MKT	Majnu ka Tila	28°41’43.6308”N	77°13’44.7168”E
3.	BVT	Buddha Vihar	28°40’28.4448”N	77°33’56.4888”E
4.	RBT	Old Delhi Railway Bridge	28°39’50.0328”N	77°14’53.8872”E
5.	GCT	Geeta Colony	28°38’59.5500”N	77°15’52.9380”E
6.	IBT	ITO Barrage	28°37’39.9532”N	77°15’10.1448”E
7.	SRT	Sarai Kale Khan	28°35’21.9480”N	77°16’15.1572”E
8.	MBT	Maharani Bagh Sub. Station	28°36’1.3464”N	77°15’39.5784”E
9.	CCT	Canal Colony	28°33’57.3522”N	77°17’42.3204”E
10.	BOT	Okhla Barrage	28˚32ˈ38.2092” N	77°18’48.1752”E

antibiotics

MDPI

Supplementary table 2- Bacterial strains used in this study with corresponding ESBLs genes and their antibiotics profiling (Siddiqui et al 2019).

Strains	**ESBLs genes**	**Antibiotics profiling**
Bacillius firmus WBT16	CTX-M15, TEM-116	AMP,AT,CAZ, CTX
E. coli MKT3	CTX-M-15, TEM-116, SHV-12	AMP,P/T,AT,CZ,CAZ,CTX,CTR
E. coli MKT25	CTX-M-15, TEM-116	AMP,A/S,P/T,AT,CZ,CAZ,CTX, CTR,PB,CIP
Shigella sonnei MKT34	CTX-M-15, TEM-116	AMP,CZ,CTX
Bacillus safensis MKT35	CTX-M-15, TEM-116	AMP,CZ,CTX,CAZ
K. quasipneumoniae MKT39	CTX-M-3, TEM-116	AMP,CZ,CTX,CAZ, A/S,P/T,AK,PB
Acinetobacter sp. MKT43	CTX-M-15, TEM-116	AMP,P/T,AT,CZ,CX,C, AZ,CTX,CTR,CIP,TR
Brachymonas chironomi MKT44	CTX-M-15, CTX-M-152, TEM-116, SHV-12	AMP,CZ,CX,CAZ,CTX,CTR,PB,CIP ,TR
Acinetobacter sp. MKT45	CTX-M-15, CTX-M-152, SHV-12	AMP,AT,CZ,CX,CAZ, CTX,CTR,CIP,TR
Acinetobacter sp. MKT46	CTX-M-15	AMP,A/S,CZ,TR
Acinetobacter sp. MKT48	CTX-M-15, CTX-M-152, SHV-42	AMP,CZ,CX,CAZ,CTX,CTR,CIP
E. coli BVT8	CTX-M-15, TEM-116	AMP,AT,CZ,CX,CAZ, CTX,CTR,PB,C
E. coli BVT20	CTX-M-15, SHV-12	AMP,A/S,P/T,AT,CZ, CX,CAZ,CTX,CTR,AK,PB,CIP,LE,TE,TR
Enterobacter cloacae BVT22	CTX-M-15, TEM-116, SHV-12	AT,CZ,CX,CAZ,CTX, PB
Enterobacter cloacae BVT29	CTX-M-15, CTX-M-152, TEM-116, SHV-12	AMP,A/S,P/T,AT,CZ, CX,CAZ,CTX,CTR,AK,PB,CIP
Enterobacter sp. BVT30	CTX-M-15	CAZ,CTX,CTR,AT
Bacillus safensis BVT32	CTX-M-152, TEM-116	CAZ,CTX,CTR,PB,C
Enterobacter cloacae BVT34	CTX-M-15, TEM-116	AMP,AT,CZ,CAZ
E. coli RBT1	CTX-M-15	AMP,A/S,P/T,AT,IPM,CZ,CX,CAZ, CTX,CTR,PB,TR
Shigella flexneri RBT20	CTX-M-15, TEM-116	AMP,P/T,AT,CZ,CX, CAZ,CTX,CTR,PB,CIP,LE,TE
Acinetobacter sp. RBT26	CTX-M-15, CTX-M-152, TEM-116	AMP,CX,CAZ,CTX, CTR,CZ,CIP,TR
Acinetobacter junii RBT31	CTX-M-15, CTX-M-152, SHV-12	AMP,CZ,CAZ,CTX, CTR,TR

Serratia marcescens RBT37	CTX-M-15, TEM-116	AT,CAZ,CTX,CTR,PB
K. pneumoniae RBT40	CTX-M-15, CTX-M-152	AT,CAZ,CTX,CTR,TR
Enterobacter cloacae GCT36	CTX-M-15	AMP,A/S,P/T,CZ,CX, CAZ,CTX,CTR,C,TE, TR
Shigella flexneri IBT7	CTX-M-15, TEM-116	AMP,A/S,P/T,AT,CZ, CX,CAZ,CTX,CTR,PB,CIP,LE
E. coli IBT13	CTX-M-15, TEM-116, SHV12	AMP,A/S,P/T,AT,CZ, CX,CAZ,CTX,CTR,AK,PB,CIP,LE,C,TE,TR
Acinetobacter junii IBT27	CTX-M-15	AMP,CZ,CTX,CTR,LE
Acinetobacter junii IBT29	CTX-M-15, TEM-116	P/T,AT,CX,CAZ,CTX,CTR,CZ,CIP, LE,C,TE,TR
Acinetobacter junii IBT30	CTX-M-15, CTX-M-152 TEM-116	CZ,CX,CAZ,CTX,CTR
Acinetobacter junii IBT31	CTX-M-15, CTX-M-152	CZ,CX,CTX,TR
Acinetobacter junii IBT33	CTX-M-15, CTX-M-152, TEM-141, SHV-12	AMP,CZ,CX,CTX,CIP
Acinetobacter junii IBT36	CTX-M-15, TEM-116	CZ,TR
Acinetobacter junii IBT39	CTX-M-15	AMP,CZ,CX,CAZ,CTX,CTR,CIP,LE,TR
Acinetobacter junii IBT40	CTX-M-15, CTX-M-152, TEM-116	CZ,CTX
E. coli SRT41	CTX-M-15, TEM-116	AMP,A/S,P/T,AT,CZ, CX,CTX,CTR
Acinetobacter calcoaceticus SRT58	CTX-M-15	AMP,P/T,CZ,CX,CAZ,CTX,CTR,CIP,C,TR
Acinetobacter sp. SRT63	CTX-M-152, TEM-116	AT,CAZ
Acinetobacter sp. SRT64	CTX-M-3	AMP,CZ,CX,CAZ,CTX,CTR,PB,CIP,TR
Acinetobacter sp. SRT65	CTX-M-15	AMP,CZ,CX,CAZ,CTX,CTR,PB,CIP,TR
E. coli MBT16	CTX-M-15	AMP,A/S,P/T,AT,CZ, CX,CAZ,CTX,CTR
E. coli MBT29	CTX-M-15	AMP,A/S,P/T,AT,CZ, CX,CAZ,CTX,CTR,AK,PB,CIP,LE,TE,TR
E. coli MBT42	CTX-M-15, TEM-116, SHV-12	AMP,CZ,TR
K. pneumoniae MBT43	CTX-M-15, TEM-116	AT,CAZ,CTX,CTR
Enterobacter cloacae MBT49	CTX-M-15, CTX-M-152, TEM-116	AT,CAZ,CTX,CTR,P/T
K. pneumoniae MBT50	CTX-M-15	AMP,P/T,AT,CZ,CX, CAZ,CTX,AK,PB,CIP
K. pneumoniae MBT51	CTX-M-15, TEM-116	AMP, AT,CAZ, CTX
K. pneumoniae MBT52	CTX-M-15, SHV-12	CAZ,CIP,LE,TE,TR
Bacillus firmus MBT57	CTX-M-15, TEM-116	AMP,AT,CZ,CAZ,CTX,CTR
K. pneumoniae MBT59	CTX-M-15, CTX-M-152 , TEM-116	AT,CAZ

E. coli CCT7	CTX-M-15, TEM-116	CAZ,CTX,CTR,AK,PB,CIP,LE,AMP,A/S,P/T,AT,IPM,CZ,TE,TR
Enterobacter cloacae CCT13	CTX-M-15	AMP,A/S,P/T,AT,IPM,CZ,CX,CAZ,CTX,CTR,PB,TE,TR
Shigella flexneri CCT27	CTX-M-15, TEM-116, SHV-12	AMP,AT,CAZ,CTX
E. coli CCT42	CTX-M-15, TEM-116	AMP,A/S,P/T,AT,CZ,CX,CAZ,CTX,CTR,AK,PB,CIP,LE,TE
E. coli CCT43	CTX-M-15, TEM-116	CAZ,CTX,CTR
E. coli CCT50	CTX-M-15	P/T,AT,IPM,CZ,CAZ,CTX,CTR,AK,PB
Kluyvera georgiana CCT51	CTX-M-15	AT,CAZ,CTX,CTR
Shigella flexneri CCT52	CTX-M-15, CTX-M-152	AT,CAZ,CTX,CTR
Acinetobacter sp. CCT53	CTX-M-15, CTX-M-152, TEM-116	CAZ,CTX,CTR
Acinetobacter sp. CCT54	CTX-M-15, TEM-116	CAZ
Acinetobacter sp. CCT59	CTX-M-15, CTX-M-152 ‘TEM-181	AMP,CZ,CX,TR
E. coli CCT64	CTX-M-15, CTX-M-152, TEM-116	AT,CAZ,CTX
Acinetobacter schindleri CCT65	CTX-M-15, CTX-M-152, TEM-116	AMP,CZ,CX,CTX,TR
Acinetobacter calcoaceticus CCT66	CTX-M-15	AMP,P/T,AT,CZ,CX,CAZ,CTX,CTR,AK,PB,TR
Kluyvera georgiana CCT69	CTX-M-152, TEM-116	AMP,P/T,AT,CZ,CX,CAZ,CTX,AK,PB,TR
E. coli BOT1	CTX-M-15	AT,CAZ,CTX,CTR
Bacillus altitudinis BOT30	CTX-M-15, TEM-116	AT,CAZ,CTR
Bacillus firmus BOT39	CTX-M-15, CTX-M-152	AMP,P/T,AT,CAZ
Acinetobacter sp. BOT40	CTX-M-15, TEM-116	AT,CAZ
Acinetobacter johnsonii BOT41	CTX-M-152	AMP,AT,CZ,CX,CAZ,CTX,CTR
Acinetobacter sp. BOT44	CTX-M-152, TEM-116	AMP,CZ,CAZ,CTX, CTR,TR
Enterobacter sp. BOT45	CTX-M-15, CTX-M-152	AT,CX,CAZ
Acinetobacter schindleri BOT46	CTX-M-15	AMP,CZ,CX,CTX
